# Defining the Mechanistic Correlates of Protection Conferred by Whole-Cell Vaccination against Pseudomonas aeruginosa Acute Murine Pneumonia

**DOI:** 10.1128/IAI.00451-20

**Published:** 2021-01-19

**Authors:** Emel Sen-Kilic, Catherine B. Blackwood, Annalisa B. Huckaby, Alexander M. Horspool, Kelly L. Weaver, Aaron C. Malkowski, William T. Witt, Justin R. Bevere, F. Heath Damron, Mariette Barbier

**Affiliations:** a Department of Microbiology, Immunology, and Cell Biology, West Virginia University School of Medicine, Morgantown, West Virginia, USA; b Vaccine Development Center, West Virginia University Health Sciences Center, Morgantown, West Virginia, USA; Washington State University

**Keywords:** humoral immune response, *Pseudomonas aeruginosa*, whole-cell vaccine, correlates of protection, vaccines

## Abstract

Pseudomonas aeruginosa is a Gram-negative pathogen that causes severe pulmonary infections associated with high morbidity and mortality in immunocompromised patients. The development of a vaccine against P. aeruginosa could help prevent infections caused by this highly antibiotic-resistant microorganism. We propose that identifying the vaccine-induced correlates of protection against P. aeruginosa will facilitate the development of a vaccine against this pathogen. In this study, we investigated the mechanistic correlates of protection of a curdlan-adjuvanted P. aeruginosa whole-cell vaccine (WCV) delivered intranasally. The WCV significantly decreased bacterial loads in the respiratory tract after intranasal P. aeruginosa challenge and raised antigen-specific antibody titers. To study the role of B and T cells during vaccination, anti-CD4, -CD8, and -CD20 depletions were performed prior to WCV vaccination and boosting. The depletion of CD4^+^, CD8^+^, or CD20^+^ cells had no impact on the bacterial burden in mock-vaccinated animals. However, depletion of CD20^+^ B cells, but not CD8^+^ or CD4^+^ T cells, led to the loss of vaccine-mediated bacterial clearance. Also, passive immunization with serum from WCV group mice alone protected naive mice against P. aeruginosa, supporting the role of antibodies in clearing P. aeruginosa. We observed that in the absence of T cell-dependent antibody production, mice vaccinated with the WCV were still able to reduce bacterial loads. Our results collectively highlight the importance of the humoral immune response for protection against P. aeruginosa and suggest that the production of T cell-independent antibodies may be sufficient for bacterial clearance induced by whole-cell P. aeruginosa vaccination.

## Abstract (precis)

Pseudomonas aeruginosa is a Gram-negative pathogen that causes severe pulmonary infections associated with high morbidity and mortality in immunocompromised patients. The development of a vaccine against P. aeruginosa could help prevent infections caused by this highly antibiotic-resistant microorganism.

## INTRODUCTION

Pseudomonas aeruginosa is a Gram-negative pathogen that causes acute and chronic severe pulmonary infections associated with high morbidity and mortality (1). The treatment of P. aeruginosa infections is often difficult due to the prevalence of antibiotic-resistant strains and the adaptability of this pathogen. P. aeruginosa infections are particularly problematic in hospitalized, immunocompromised, elderly, and cystic fibrosis (CF) patients (2). Based on the 2018 Cystic Fibrosis Foundation report, by the time CF patients reach adulthood, almost 70% are infected with P. aeruginosa (3). Early eradication of P. aeruginosa in life before colonization may help prevent infections caused by this bacterium (4). Therefore, numerous researchers have proposed over the years that developing a protective immune response against P. aeruginosa early in life through vaccination can help prevent infections caused by this bacterium. This preventative strategy could increase the quality and longevity of life in susceptible patients and provide solutions where antibiotic treatments are failing.

The development of novel immunotherapies and vaccines against P. aeruginosa has been extensively studied, and numerous experimental vaccine candidates have been evaluated (2, 5). Among them, whole-cell and live-attenuated P. aeruginosa mucosal vaccines were shown to be protective in animal models (6–12). Unfortunately, while early clinical trials using whole-cell P. aeruginosa vaccines were promising (13–15), there were concerns about efficacy against different P. aeruginosa serotypes and potential toxicity (16, 17). Subunit vaccines based on virulence factors of P. aeruginosa, such as flagella (18–21), lipopolysaccharide (LPS) (22–25), and outer membrane proteins OprF to OprI (26–31), have also been developed and tested in clinical trials, but to this day, none have been approved for human use. To generate novel treatment options and vaccine strategies against P. aeruginosa and increase their chances of clinical success, it is essential to first thoroughly understand vaccine-induced immunity against P. aeruginosa in preclinical models.

During acute P. aeruginosa infections, innate immune responses play an important role in bacterial clearance (32). Neutrophils are recruited to infected lungs during pneumonia and are considered the main effector cell population of the innate response against P. aeruginosa (33). In the context of vaccine-induced protection, P. aeruginosa*-*specific antibodies are an important component of the adaptive immune response (5). Passively acquired polyclonal or monoclonal antibodies generated to various outer membrane proteins and virulence factors protect against P. aeruginosa (2, 5, 17). In addition, CD4^+^ T cells especially play a role against P. aeruginosa in animals vaccinated with bivalent flagellin and live-attenuated strains in the absence of LPS O antigen-specific opsonophagocytic antibodies (9, 10, 34, 35). These studies have shed light on the importance of the various individual mechanisms involved in the innate and adaptive immune responses against P. aeruginosa. However, their relative contributions to protection against P. aeruginosa during vaccination remain to be determined.

In this study, we identified the relative role of vaccine-induced humoral and T cell-mediated immunity against P. aeruginosa during acute murine pneumonia by using a heat-inactivated whole-cell P. aeruginosa vaccine. To generate immunity at the site of infection, we administered the vaccine intranasally (36, 37). We used curdlan as an adjuvant to induce a Th1/Th17 immune response (38, 39). We observed that depletion of CD20^+^ B cells, but not CD8^+^ or CD4^+^ T cells, prior to whole-cell vaccine (WCV) priming resulted in the loss of bacterial clearance and that the whole-cell vaccine-mediated humoral immune response was the most critical component of immunity. We expect that this work will provide valuable insights into the mechanisms underlying whole-cell vaccine-mediated protection and inform the development of future vaccines against P. aeruginosa.

## RESULTS

### Depletion of CD4^+^ and CD8^+^ T cells during WCV priming does not affect bacterial burden or lung edema during acute pneumonia.

**FIG 1:**
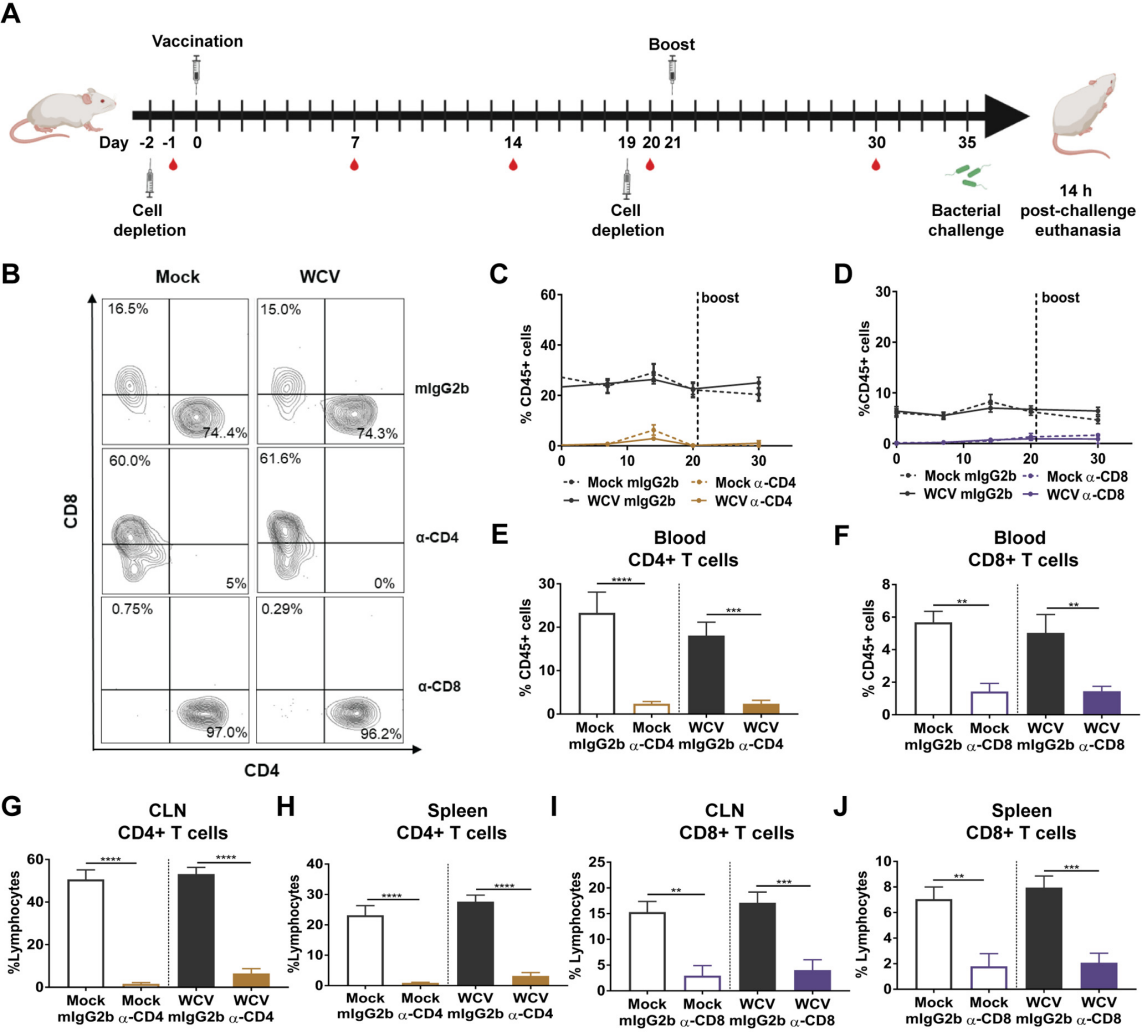
CD4^+^ and CD8^+^ T cell depletion during vaccination and challenge. (A) Schematic representation of the vaccination/challenge timeline (B) Representative contour plots showing the proportion of CD4^+^ and CD8^+^ T cell populations in CD3e^+^ CD45^+^ leukocytes at day 20 postvaccination. (C and D) The proportions of CD3e^+^ CD4^+^ T cells (*n* = 4 or 5 per group) (C) and CD3e^+^ CD8^+^ T cells in CD45^+^ cells during vaccination in the blood (*n* = 3 to 5 per group) (D). (E, G, and H) The proportions of CD3e^+^ CD4^+^ T cells in blood (E), cervical lymph node (CLN) (G), and spleen (H) CD45^+^ leukocytes in mock-vaccinated or whole-cell-vaccinated mice treated with IgG2b isotype control or anti-CD4 antibodies at 14 h p.c. (*n* = 8 to 10 per group). (F, I, and J) The proportions of CD3e^+^ CD8^+^ T cells in blood (F), cervical lymph node (I), and spleen (J) CD45^+^ leukocytes in mock-vaccinated or whole-cell-vaccinated mice treated with IgG2b isotype control or anti-CD8 at 14 h p.c. (*n* = 5 to 9 per group). Data represent those from four independent experiments. ANOVA followed by Tukey’s multiple-comparison test was used for statistical analysis. Error bars indicate SEMs. The asterisks show statistical significance as follows: **, *P ≤ *0.01; ***, *P ≤ *0.001; and ****, *P ≤ *0.0001.

**FIG 2:**
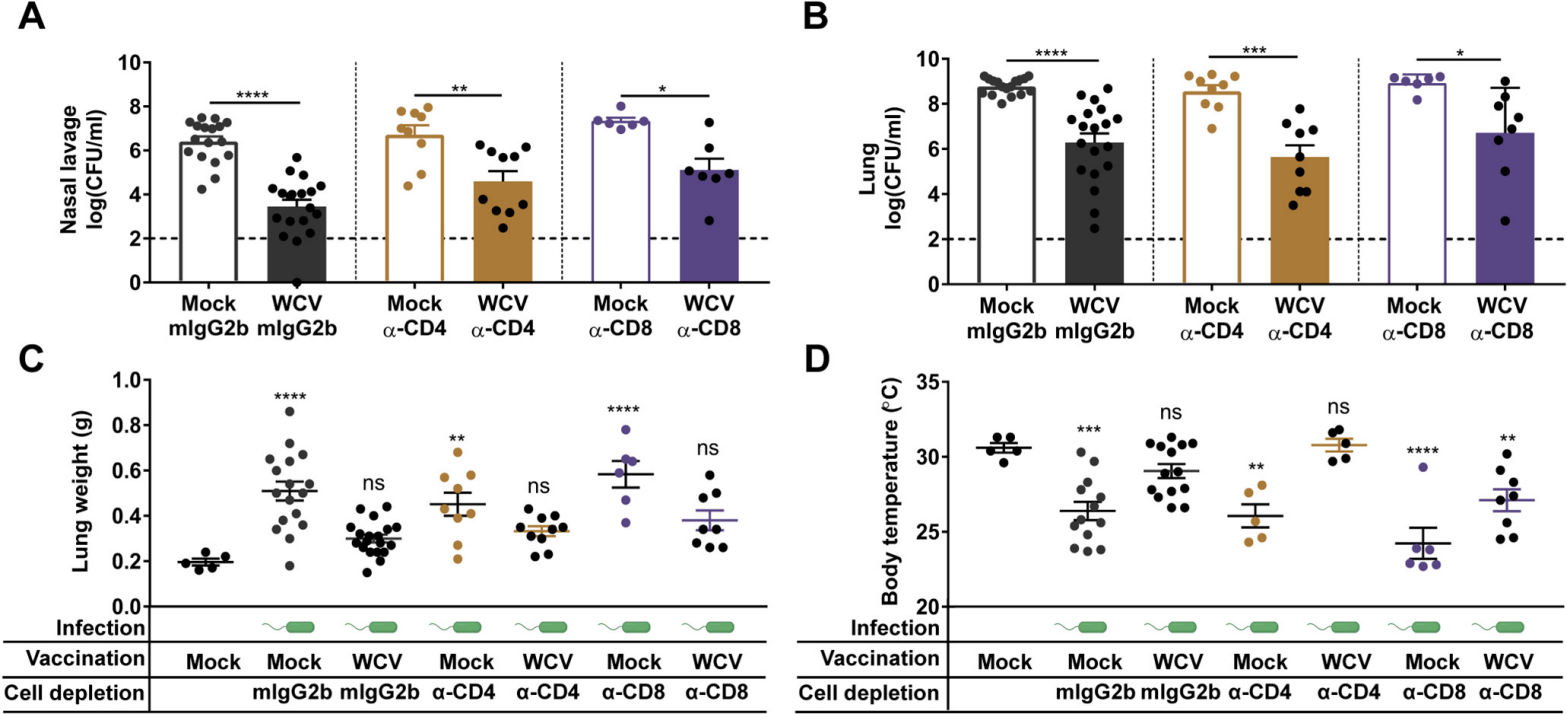
CD4^+^ or CD8^+^ T cell depletion does not affect the bacterial burden and lung edema during acute pneumonia in WCV mice. (A and B) Nasal lavage (A) and lung (B) bacterial loads of vaccinated and challenged groups at 14 h p.c. (*n* = 6 to 17 per group). (C and D) Lung weights (C) and body temperatures (D) of mice groups at 14 h p.c. (*n* = 5 to 17 per group). Data represent those from four independent experiments. Dashed lines represent the lower limit of detection. ANOVA followed by Tukey’s multiple-comparison test was used for statistical analysis. Error bars indicate SEMs. The asterisks show statistical significance as follows: *, *P ≤ *0.05; **, *P ≤ *0.01; ***, *P ≤ *0.001; and ****, *P ≤ *0.0001.

CD4^+^ T cells induce specific cytokines to coordinate innate and adaptive immune responses that promote bacterial clearance (34, 40) and provide support for antigen-specific B cell maturation, proliferation, and class switching (41). On the other hand, CD8^+^ T cells also play a role in bacterial clearance by secreting cytokines, producing cytotoxic granules, and direct killing of infected cells (42, 43). To understand the role of CD4^+^ and CD8^+^ T cells in vaccine-mediated protection against P. aeruginosa, we depleted CD4^+^ and CD8^+^ T cells using monoclonal antibodies (MAbs) prior to WCV priming (Fig. 1A). We hypothesized that depletion of either CD4^+^ or CD8^+^ T cells would impair the murine immune response to P. aeruginosa in both naive and vaccinated animals. Using flow cytometry, cell depletions were confirmed in blood during vaccination and challenge, as well as in secondary lymphoid organs after challenge. At day 1 postdepletion, >98% of CD4^+^ T cells and CD8^+^ T cells were depleted in mice receiving anti-CD4 and anti-CD8 MAbs, respectively (Fig. 1C and D). Throughout the course of vaccination, both CD8^+^ and CD4^+^ T cell populations remained depleted from blood in mice that received anti-CD8 and anti-CD4 treatment (Fig. 1B to D). After challenge, we observed significant reductions in frequencies of CD4^+^ and CD8^+^ T cell populations in the blood, spleen, and cervical lymph nodes of anti-CD4- and anti-CD8-treated mice compared to those in isotype control mice (Fig. 1E to J). To evaluate the effect of cell depletion on the response to infection, we measured the bacterial burdens in the lungs and nares 14 h postchallenge (p.c.). We did not observe significant differences in the bacterial burdens in the nares and the lungs of mock-vaccinated mice, which received anti-CD4, anti-CD8, or anti-IgG2b isotype control treatments (Fig. 2A and B). In mice that received the isotype control, we observed that vaccination with the WCV led to a significant decrease in bacterial burden in the nares and lungs compared to those in the mock-vaccinated group, which demonstrates that the intranasal WCV is protective against P. aeruginosa acute pneumonia in this model (Fig. 2A and B). Interestingly, mice vaccinated with the WCV in which CD4^+^ or CD8^+^ T cells were depleted also had a significantly lower number of bacteria in the nares and the lungs 14 h p.c. than CD4^+^ or CD8^+^ T cell-depleted mock-vaccinated mice (Fig. 2A and B). These results suggest that neither CD4^+^ T cell nor CD8^+^ T cell depletion prior to vaccination and boost affects bacterial clearance in WCV mice.

Acute pneumonia caused by P. aeruginosa is associated with lung injury and pulmonary edema (44). As an indicator of lung edema, lung weights were measured in each group. We observed that lung weights increased in response to challenge in mock-vaccinated mice compared to those in noninfected mock-vaccinated mice (adjuvant only), regardless of the presence of circulating CD4^+^ or CD8^+^ T cells (Fig. 2C). We did not observe an increase in the weight of the lung in mice vaccinated with the WCV, regardless of the depletion of CD4^+^ or CD8^+^ T cells (Fig. 2C). These results demonstrate that neither CD4^+^ T cell nor CD8^+^ T cell depletion affected the degree of lung edema in mock-vaccinated or WCV group mice.

Mouse body temperature is another physiological parameter associated with lung infection caused by P. aeruginosa (45). We observed a significant decrease in the surface body temperature of mock-vaccinated challenged mice compared to that of the noninfected mock-vaccinated control (Fig. 2D). In contrast, the temperature of the WCV group of mice treated with isotype control or anti-CD4 antibodies and challenged remained similar to the temperature of noninfected mock-vaccinated control mice (Fig. 2D). Surprisingly, the body temperature of CD8^+^ T cell-depleted WCV group mice decreased compared to that of noninfected mock-vaccinated animals (Fig. 2D). Overall, the bacterial burden, lung weight, and body temperature data indicate that mice vaccinated with the WCV were protected against P. aeruginosa infection. In addition, the protection conferred by whole-cell vaccination did not depend on the presence of circulating CD4^+^ or CD8^+^ T cells.

### Depletion of B cells abrogates WCV-mediated protection.

**FIG 3:**
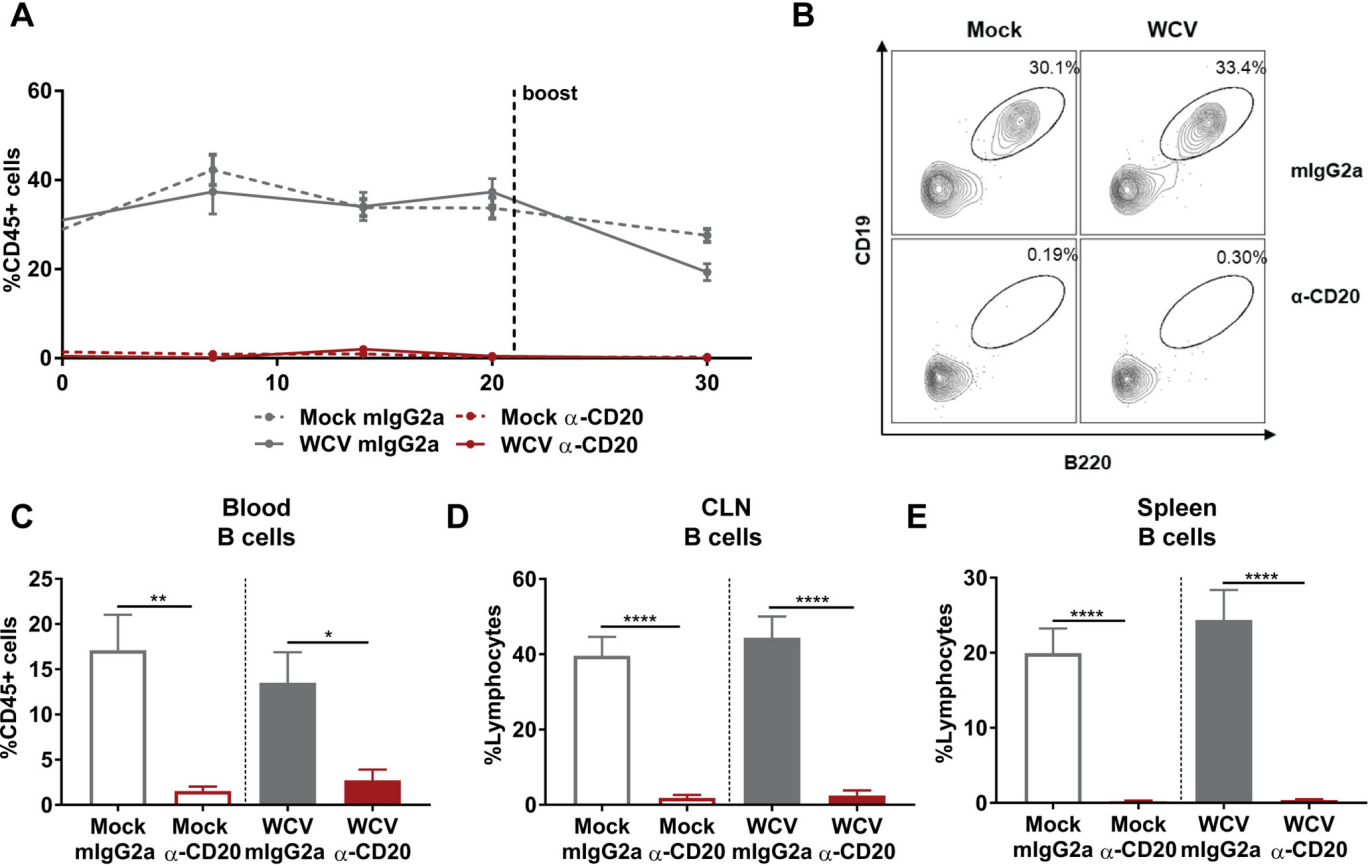
CD20^+^ cell depletion during vaccination and challenge. (A) The proportion of B220^+^ CD19^+^ B cells in CD45^+^ leukocytes during vaccination in the blood (*n* = 4 or 5 per group). (B) Representative contour plots of B220^+^ CD19^+^ B cells showing the proportion of these cells in CD45^+^ leukocytes at day 20 postvaccination. (C to E) The proportions of B220^+^ CD19^+^ B cells in blood (C), cervical lymph nodes (CLN) (D), and spleen (E) CD45^+^ leukocytes in mock-vaccinated or whole-cell-vaccinated mice treated with IgG2a isotype control or anti-CD20 antibodies at 14 h p.c. (*n* = 9 or 10 per group). Data represent those from two independent experiments. ANOVA followed by Tukey’s multiple-comparison test was used for statistical analysis. Error bars indicate SEMs. The asterisks show statistical significance as follows: *, *P ≤ *0.05; **, *P ≤ *0.01; and ****, *P ≤ *0.0001.

**FIG 4:**
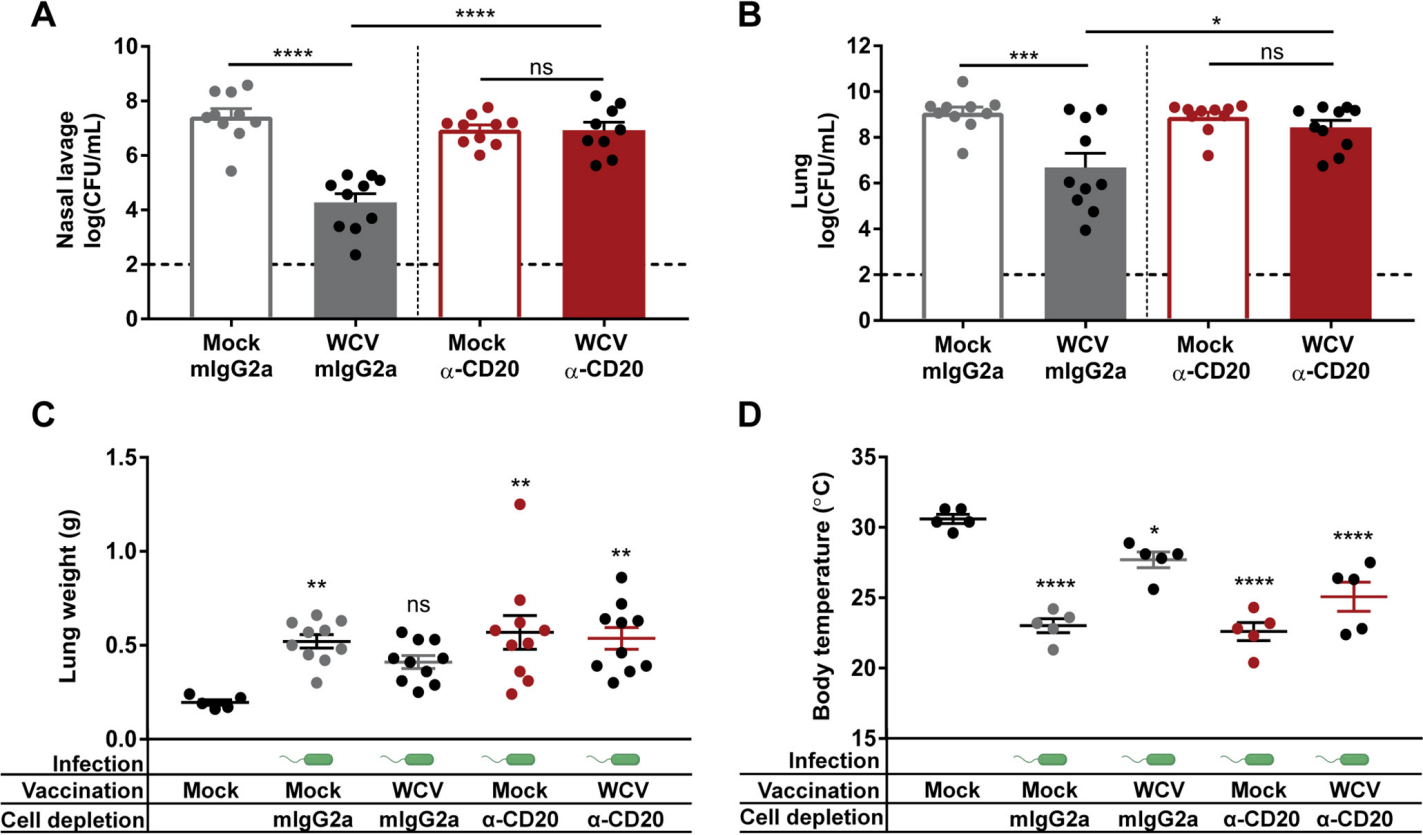
B cell depletion prior to WCV priming abrogates the protection against P. aeruginosa during acute pneumonia. (A and B) Nasal lavage (A) and lung (B) bacterial loads of vaccinated and challenged groups at 14 h p.c. (*n* = 9 or 10 per group). (C and D) Lung weights (C) and body temperatures (D) of mice groups at 14 h p.c. (*n* = 5 to 10 per group). Dashed lines represent the lower limit of detection. Data represent those from two independent experiments. ANOVA followed by Tukey’s multiple-comparison test was used for statistical analysis. Error bars indicate SEMs. The asterisks show statistical significance as follows: *, *P ≤ *0.05; **, *P ≤ *0.01; ***, *P ≤ *0.001; and ****, *P ≤ *0.0001.

To understand the role of B cells in whole-cell vaccine-mediated immunity against P. aeruginosa, we performed B cell depletion experiments prior to WCV priming using an anti-CD20 MAb (Fig. 1A). CD20 is expressed on immature, mature, germinal center, and memory B cells but not on plasma cells, plasmablasts, or pro-B cells (46–48). We depleted B cells prior to vaccination and boosting and monitored the presence of circulating B cells over time. At day 1 post-CD20 depletion, we observed that the proportion of CD19^+^ B220^+^ B cells in blood was reduced from average of 28% to 1%, which corresponds to a depletion of >96% (Fig. 3A). B cell depletion was sustained throughout the duration of the experiment in the blood (Fig. 3A to C). B cells were also depleted in the cervical lymph nodes (CLN) and the spleens of anti-CD20-treated groups after challenge (Fig. 3D and E). To evaluate whether depletion of B cells during vaccination plays a role in protection during acute pneumonia caused by P. aeruginosa, the WCV group and mock-vaccinated mice that received anti-CD20 or IgG2a isotype control MAbs were challenged at day 35. At 14 h p.c., the bacterial burden in the nasal lavage samples and lung homogenates was determined. We did not observe changes in the bacterial loads of nasal lavage samples and lung homogenates of mock-vaccinated mice that received anti-CD20 or isotype control MAbs (Fig. 4A and B). These data indicate that the depletion of B cells does not impact the murine response to challenge in mock-vaccinated animals. WCV group mice that received isotype control MAb had a significantly lower number of bacteria in the lungs and nasal lavage samples than did isotype control-treated mock-vaccinated mice (Fig. 4A and B). However, B cell-depleted WCV group mice had a significantly higher number of bacteria in the lungs and nasal lavage samples than did isotype control-treated WCV group mice (Fig. 4A and B).

As expected, we observed that the lung weights of mock-vaccinated mice increased in response to challenge compared to that of the noninfected control, regardless of the presence of circulating B cells (Fig. 4C). However, depletion of B cells in WCV group mice also resulted in a significant increase in lung weight compared to that of the noninfected control (Fig. 4C). While the body surface temperature decreased in response to challenge in all groups, the decrease observed in B cell-depleted mice vaccinated with the WCV was more modest (Fig. 4D). Overall, these results indicate that WCV group mice with depleted B cells have a more severe P. aeruginosa infection than do isotype control-treated WCV group mice.

### B cell depletion results in a decrease in antigen-specific antibodies which negatively correlates to bacterial loads in WCV group mice.

**FIG 5:**
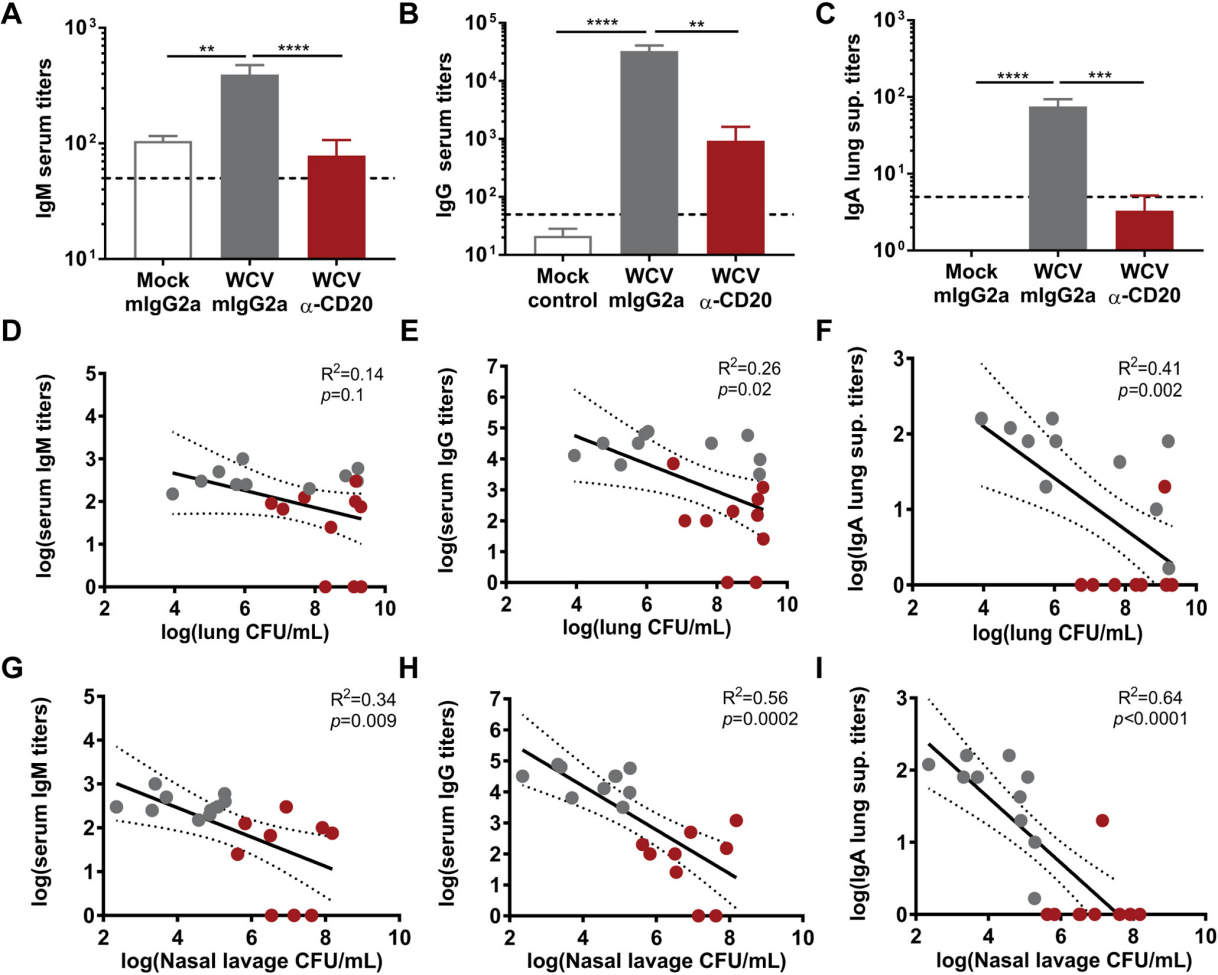
Depletion of B cells during P. aeruginosa whole-cell vaccination reduces antigen-specific antibody titers. (A to C) P. aeruginosa-specific IgM (A) and IgG (B) serum antibody titers and IgA lung supernatant antibody titers (C) of vaccinated and challenged mouse groups (*n* = 9 or 10 per group). Dashed lines represent the limit of detection. Kruskal-Wallis test with Dunn’s multiple comparison was used for statistical analysis. (D to F) Correlation of lung CFU per milliliter with serum IgM (D), serum IgG (E), and lung supernatant IgA (F) titers in whole-cell-vaccinated animals. (G to I) Correlation of nasal lavage CFU per milliliter with serum IgM (G), serum IgG (H), and lung supernatant IgA (I) titers in whole-cell-vaccinated animals. Gray dots represent whole-cell-vaccinated IgG2a isotype control-treated mice, and red dots represent WCV anti-CD20-treated mice. Each dot represents data from one individual mouse. The *R*^2^ and *P* values for each correlation were calculated using linear regression analyses. Error bars indicate SEMs. The asterisks show statistical significance as follows: **, *P ≤ *0.01; ***, *P ≤ *0.001; and ****, *P ≤ *0.0001.

The primary role of B cells during vaccination is to produce antigen-specific antibodies (49). To observe the effect of anti-CD20 MAb treatment on vaccine-induced P. aeruginosa-specific antibody production, we measured serum and lung supernatant antibody titers using enzyme-linked immunosorbent assay (ELISA). The isotype control-treated WCV group mice showed significant anti-P. aeruginosa serum IgM, IgG, and IgA lung supernatant titers compared to those of isotype control-treated mock-vaccinated mice (Fig. 5A to C). Anti-P. aeruginosa serum IgM, IgG, and IgA lung supernatant titers were significantly lower in B cell-depleted WCV group mice than in isotype control-treated WCV group mice (Fig. 5A to C). To understand whether the loss of the antigen-specific immune response correlates with bacterial clearance, we compared the lung bacterial burdens to antibody titers. We observed a negative correlation between serum IgM and bacterial loads in the nasal lavage samples but not in lung homogenates (Fig. 5D and G). Both anti-P. aeruginosa serum IgG and lung supernatant IgA titers negatively correlated with bacterial loads in the lungs and nasal lavage samples (Fig. 5E, F, H, and I). The results obtained demonstrate that depletion of B cells prior to WCV priming negatively affects the production of P. aeruginosa-specific antibodies, which are correlated with bacterial clearance.

### Polyclonal serum from WCV group mice is sufficient for bacterial clearance of P. aeruginosa.

**FIG 6:**
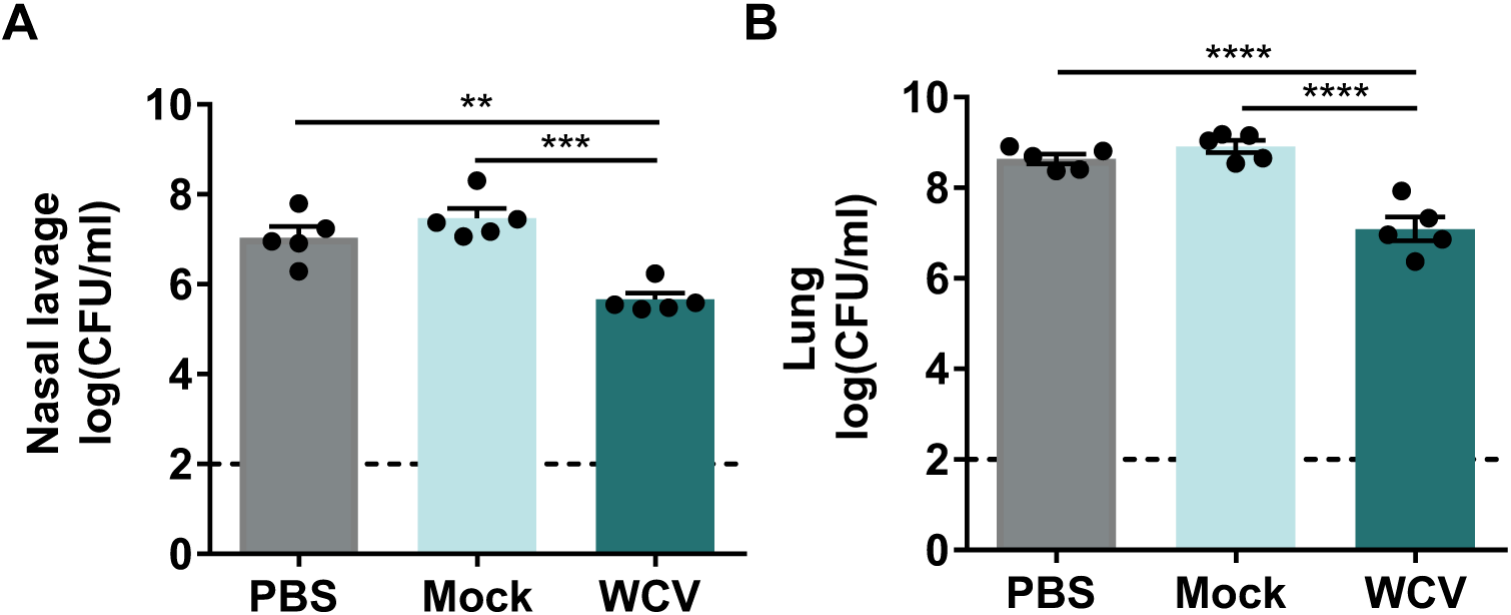
Preopsonization of P. aeruginosa with sera WCV group mice decreases bacterial burden in lungs and nasal lavage samples. Shown are bacterial burdens in nasal lavage (A) and lung samples (B) of mice infected with 10^7^ CFU/dose of P. aeruginosa preopsonized with mock sera, WCV mouse group sera, or nonopsonized PBS control at 16 h p.c. (*n* = 5 per group). ANOVA followed by Tukey’s multiple-comparison test was used for statistical analysis. Error bars indicate SEMs. The asterisks show statistical significance as follows: **, *P ≤ *0.001; ***, *P ≤ *0.001; and ****, *P ≤ *0.0001.

**FIG 7:**
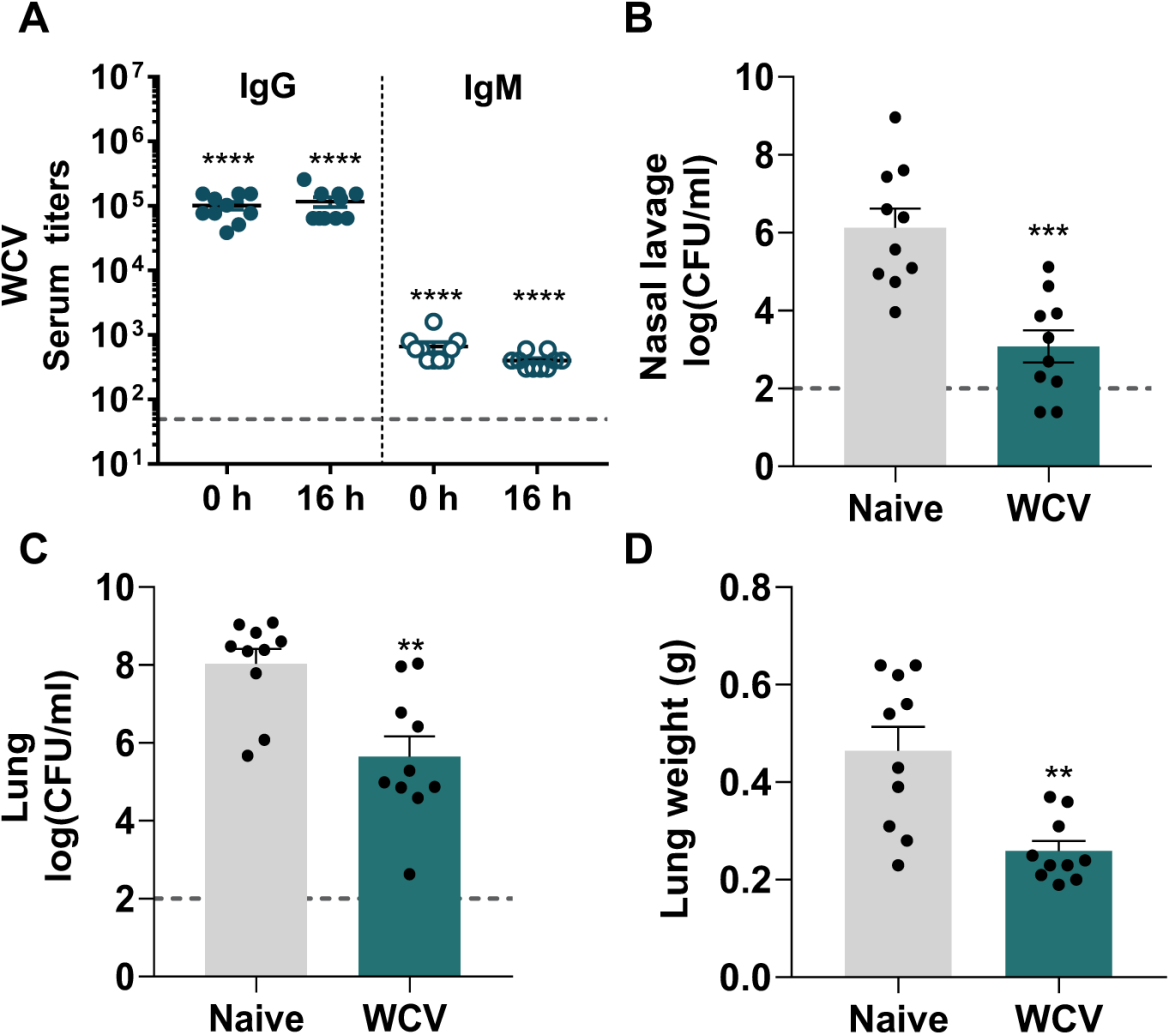
Passive immunization alone is sufficient to decrease bacterial loads during P. aeruginosa acute pneumonia. (A) P. aeruginosa-specific serum IgG and IgM titers at 0 h and 16 h p.c. of animals passively immunized with WCV. For statistical analysis, a one-sample *t* test comparing the data to the value of the detection was performed. (B to D) The bacterial burdens of nasal lavage (B) and lung samples (C) and lung weights (D) of mice passively immunized with sera from WCV group and naive mice at 16 h p.c. (*n* = 10 per group). Data represent those from two independent experiments. Dashed lines represent the limit of detection. For comparisons, unpaired two-tailed Student’s *t* test was used. Error bars indicate SEMs. The asterisks show statistical significance as follows: **, *P ≤ *0.01; ***, *P ≤ *0.001; and ****, *P ≤ *0.0001.

To determine if antibodies generated in response to WCV participate in bacterial clearance during P. aeruginosa acute pneumonia, we performed preopsonization and passive-immunization experiments. We observed that the bacterial burdens in the nares and lungs of mice infected with P. aeruginosa preopsonized with sera from WCV group mice were significantly decreased compared to those of mice infected with P. aeruginosa phosphate-buffered saline (PBS) control or preopsonized with the mock sera (Fig. 6). To determine if a similar effect could be observed using circulating antibodies, we performed passive immunization. We collected the sera from WCV group and naive immunized mice at day 35. Pooled sera were administered to 5-week-old CD1 mice intraperitoneally for passive immunization 1 h prior to challenge with P. aeruginosa. Control mice received serum from naive mice that did not contain anti-P. aeruginosa antibodies (data not shown). The presence of anti-P. aeruginosa IgM and IgG in mice passively immunized with sera from WCV group mice were detected at both 0 h and 16 h p.c. (Fig. 7A). We observed that passive immunization with sera from WCV group mice significantly decreased bacterial burden and lung edema compared to those in the naive serum control group (Fig. 7B to D). These data show that serum antibodies from WCV group mice alone contribute to bacterial clearance during acute P. aeruginosa infection.

### Depletion of CD4^+^ T cells is associated with changes in antigen-specific antibody isotype switching.

**FIG 8:**
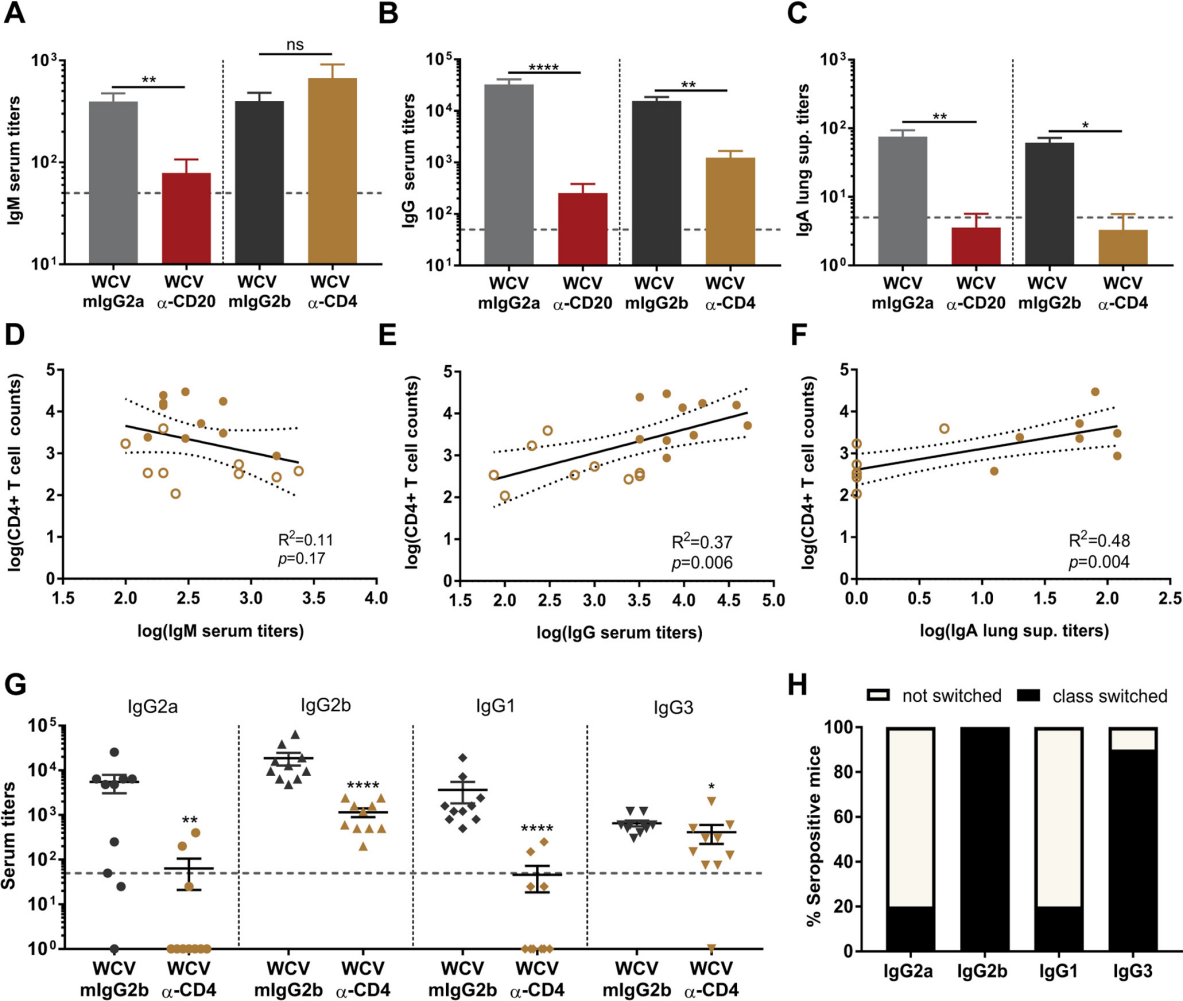
CD4^+^ T cell depletion prior to WCV priming affects T cell-dependent isotype class switching in WCV. (A to C) P. aeruginosa-specific IgM (A) and IgG (B) serum antibody titers and IgA lung supernatant antibody titers (C) of vaccinated and challenged mice (*n* = 5 to 19 per group). Data represent those from six independent experiments. Kruskal-Wallis test with Dunn’s multiple-comparison test for statistical analysis was used. (D to F) Correlation of CD4^+^ T cells blood counts with P. aeruginosa-specific serum IgM (D) and serum IgG (E) and lung supernatant IgA titers (F) in whole-cell-vaccinated animals. Yellow filled dots represent whole-cell-vaccinated IgG2b isotype control mice (WCV mIgG2b). Yellow circles represent whole-cell-vaccinated anti-CD4-treated mice (WCV α-CD4). Each circle/dot represents values from one individual mouse. The *R*^2^ and *P* values for each correlation were calculated using linear regression analyses. (G) P. aeruginosa-specific serum IgG2a, IgG2b, IgG1, and IgG3 antibody titers (*n* = 10 per group). Mann-Whitney U test was used for comparisons. Error bars indicate SEMs. Limits of detection are shown with dashed lines. The asterisks show statistical significance as follows: *, *P ≤ *0.05; **, *P ≤ *0.01; and ****, *P ≤ *0.0001. (H) Percentage of anti-P. aeruginosa IgG subtype response above the limit of detection in whole-cell-vaccinated anti-CD4-treated mice.

CD4^+^ T cells play a critical role in immunoglobulin class switching by providing critical help to germinal center B cells during vaccination (41). While we observed that antibodies participate in bacterial clearance, depletion of CD4^+^ T cells during WCV priming did not affect bacterial burden during acute pneumonia. As a result, we hypothesized that B cell isotype class switching triggered by CD4^+^ T cells is not required for protection against P. aeruginosa. To test this hypothesis, we compared the anti-P. aeruginosa serum IgG and IgM and lung supernatant IgA titers in mice that received anti-CD20, anti-CD4, and isotype controls. We observed that depletion of CD4^+^ T cells did not affect the production of antigen-specific IgM, while the depletion of B cells significantly reduced the antigen-specific IgM titers in WCV group mice (Fig. 8A). As expected, depletion of CD4^+^ T cells prior to WCV priming significantly decreased antigen-specific serum IgG and lung supernatant IgA compared to titers in mice given the IgG2b isotype control (Fig. 8B and C). The anti-P. aeruginosa serum IgG and lung supernatant IgA titers were similar in B cell-depleted and CD4^+^ T cell-depleted WCV group mice (Fig. 8B and C). We did not observe any differences in anti-P. aeruginosa-specific antibody titers in CD8^+^ T cell-depleted WCV group mice compared to the WCV control (data not shown). P. aeruginosa-specific serum IgG and lung supernatant IgA titers significantly correlated with the number of CD4^+^ T cells present in the serum, but this was not the case for serum IgM titers (Fig. 8D to F). The depletion of CD4^+^ T cells also significantly reduced anti-P. aeruginosa IgG1, IgG2a, IgG2b, and IgG3 serum responses relative to those in the isotype control-treated WCV group mice (Fig. 8G). We observed that only 20% of anti-CD4-treated WCV group mice were able to produce an anti-P. aeruginosa-specific IgG2a and IgG1 response above the limit of detection, while 100% and 90% of mice were able to elicit an anti-P. aeruginosa IgG2b and IgG3 response in serum (Fig. 8G and H). These results show that the depletion of CD4^+^ T cells decreased class switching of P. aeruginosa-specific antibodies to IgA, IgG2a, and IgG1. Overall, the results obtained demonstrate that the depletion of B cells diminished the production of P. aeruginosa-specific antibodies, while the depletion of CD4^+^ T cells mainly had an impact on T cell-dependent P. aeruginosa-specific antibody production in WCV group mice.

## DISCUSSION

Development of a vaccine against P. aeruginosa has been challenging due to the complex pathogenesis of the bacterium and the lack of a complete understanding of host immune responses required for protection (16, 17). To characterize the host immune response required for bacterial clearance, we used a protective intranasal heat-inactivated whole-cell vaccine as a model (11). By performing systematic depletion of B cells and CD4^+^ and CD8^+^ T cells, we demonstrated that the humoral immune response elicited by this vaccine is one of the main vaccine-induced mechanistic correlates of protection against P. aeruginosa.

In this study, we observed that the depletion of CD4^+^ T cells and CD8^+^ T cells does not impact bacterial burden or lung edema in whole-cell-vaccinated animals at 14 h p.c. (Fig. 2). These data are in line with the work of others showing that depletion of CD8^+^ T cells does not reduce the protective immunity acquired by vaccination with X-ray-irradiated live-attenuated P. aeruginosa (9). At first glance, the results obtained on the role of CD4^+^ T cells on vaccine-mediated protection against P. aeruginosa seem to contradict previous studies. Various authors have identified the importance of CD4^+^ T cells in live-attenuated or killed whole-cell P. aeruginosa vaccines either by transferring CD4^+^ T cells from killed whole-cell P. aeruginosa-immunized rats to naive rats (50) or by depleting CD4^+^ T cells and challenging with LPS heterologous strains in live-attenuated P. aeruginosa-vaccinated mice (9, 10). However, these published observations suggest that CD4^+^ T cells primarily play a role in models in which the challenged animals do not have opsonic antibodies against the serotype of the strain used for challenge (9, 10). In this study, preparation of the WCV and challenge were performed with an LPS-homologous strain, which is a major difference from previously published works. The role of CD4^+^ T cells for protection against LPS-heterologous strains in whole-cell-vaccinated mice needs to be further determined in future studies.

While there is extensive evidence supporting the role of antibodies in protection against P. aeruginosa acute pneumonia, the role of B cells is not clearly defined. We observed that depleting B cells during vaccination and boost led to an increase in bacterial burden in whole-cell-vaccinated and challenged mice (Fig. 4A and B). One of the primary roles of B cells is to generate antigen-specific antibodies. Antigen-specific antibodies raised in response to vaccination can help prevent infection and are a correlate of protection for many commercially available vaccines against pathogenic bacteria (51). Production of IgM is often followed by class switching and production of IgG antibodies (52), which play an essential role in bacterial clearance by promoting toxin neutralization, opsonization, and complement-mediated killing of bacteria (53, 54). On mucosal surfaces, in addition to IgG, secretion of IgA antibodies serves as one of the first lines of defense against these pathogens, providing local immunity and inhibiting the adherence of pathogens on mucosal epithelial cells (54, 55). During respiratory infections, P. aeruginosa colonizes the respiratory tract (56), and both mucosal and systemic vaccine-induced immune responses have been shown to be elicited against this pathogen in preclinical studies (17). Mucosal immunization with killed P. aeruginosa whole-cell vaccine induces anti-P. aeruginosa IgG, IgM, and IgA antibody titers (50). Similarly, we observed that intranasal vaccination with heat-inactivated whole-cell P. aeruginosa elicits anti-P. aeruginosa-specific serum IgM, IgG, and lung supernatant IgA antibody titers that participate in both mucosal and systemic humoral immune responses against P. aeruginosa (Fig. 5A to C). Depleting B cells during whole-cell vaccination lead to a significant decrease in anti-P. aeruginosa specific serum IgM, IgG, and lung supernatant IgA antibody titers and negatively correlated with bacterial loads in the nares (Fig. 5). IgM, IgG, and IgA antibodies participate in bacterial clearance through various mechanisms, including phagocytosis and complement deposition. Further studies are required to identify the exact mechanism of clearance provided by these immunoglobulins in this model of vaccination and challenge. Interestingly, only serum IgG and lung supernatant IgA titers were significantly correlated with the bacterial loads in the lungs (Fig. 5E and F). Passive immunization and preopsonization experiments using sera from whole-cell-vaccinated mice significantly decreased the bacterial burden and lung edema, suggesting that serum antibodies alone are sufficient to protect against this pathogen (Fig. 6 and 7). These results support the findings of previous studies showing that passive immunization with serum antibodies or monoclonal antibody therapies specific to virulence factors such as PcrV, lipopolysaccharide (LPS) O antigen, Psl, flagella, alginate, and outer membrane proteins such as OprF and OprI can protect against P. aeruginosa (2, 35, 57–64).

The opsonic antibodies generated by live-attenuated or whole-cell P. aeruginosa vaccinations are shown to be directed in part against LPS (10, 13). Anti-LPS antibodies can assist with bacterial clearance (61). However, the serotype variability of clinically relevant P. aeruginosa isolates (65), as well as the selection of mutants with a truncation of the O antigen during chronic respiratory infections such as those observed in CF patients (65–67) raise concern regarding their potential use for clinical vaccine applications. It is important to note that the production of IgM, IgG2b, and IgG3 antibodies can be elicited in response to highly repetitive structures, such as LPS, in a T cell-independent manner (68–70). In CD4^+^ T cell-depleted whole-cell-vaccinated mice, P. aeruginosa-specific circulating IgG antibodies were significantly reduced, while the levels of P. aeruginosa specific IgM antibodies were unchanged (Fig. 8A and B). Interestingly, we observed that these mice were still protected against P. aeruginosa, which suggests that T cell-dependent isotype switching is not required for bacterial clearance during challenge with an LPS-homologous strain (Fig. 2A and B). IgM antibodies can efficiently activate the complement system and play a role in neutralizing the pathogen (52, 68). In line with our findings, patients with selective IgM deficiency (SIGMD) or splenectomy are prone to infections caused by microbial organisms, including P. aeruginosa (71, 72). Our data suggest that IgM antibodies play an important role in WCV-induced protection against P. aeruginosa and that the use of IgM anti-P. aeruginosa antibody therapy such as the anti-LPS O antigen IgM monoclonal antibody (KBPA101, panobacumab) (61, 73, 74) can be a potential treatment against P. aeruginosa. In addition, while anti-P. aeruginosa IgG antibodies were significantly reduced in CD4^+^ T cell-depleted whole-cell-vaccinated mice, we observed that IgG2b and IgG3 serotypes were still present, albeit reduced in CD4^+^ T cell-depleted whole-cell-vaccinated mice. These results suggest that T cell-independent antibody production elicited by whole-cell vaccination may be sufficient to facilitate the clearance of P. aeruginosa during acute pneumonia when mice are challenged with an LPS-homologous strain.

This work focused on identifying the mechanistic correlates of protection provided by an intranasal heat-killed whole-cell vaccine. We observed that depletion of B cells but not CD8^+^ or CD4^+^ T cells during whole-cell vaccine priming resulted in the loss of protection against P. aeruginosa challenge with an LPS-homologous strain. We also observed that T cell-independent IgG and IgM antibody production by B cells may potentially be sufficient for whole-cell-vaccine-induced protection. Our data suggest that the vaccine-mediated humoral immune response is a critical component of whole-cell-vaccine-induced immunity against P. aeruginosa challenge with an LPS-homologous strain in the acute murine pneumonia model. We anticipate that this study will establish a blueprint of vaccine-mediated protection against P. aeruginosa and facilitate the development of future subunit vaccines.

## MATERIALS AND METHODS

### Bacterial strains and growth conditions.

P. aeruginosa PAO1 (Michael L. Vasil, University of Colorado) was used for vaccination and challenge experiments. For challenge experiments, P. aeruginosa was grown from a frozen stock on lysogeny agar (LA; Miller formulation) overnight at 37°C. A single colony from the LA plate was grown in lysogeny broth (LB; Miller formulation) overnight and diluted 1:100 in 3 ml of fresh LB for exponential growth for 4 h unless otherwise specified.

### Vaccine preparation and murine immunization.

To prepare the whole-cell P. aeruginosa vaccine used in this study, P. aeruginosa PAO1 was grown overnight at 37°C on *Pseudomonas* isolation agar (PIA; Becton, Dickinson; 292710). Bacteria were resuspended in endotoxin-free Dulbecco’s phosphate-buffered saline (PBS) (Thermo Fisher; TMS012A) and inactivated for 1 h at 60°C. The heat-treated bacteria were plated to confirm that the bacteria were killed. Each dose of WCV contained 1 × 10^8^ to 2 × 10^8^ CFU and 200 μg of curdlan (InvivoGen; 54724-00-4) in PBS. The adjuvant-only control (mock) contained 200 μg of curdlan in PBS. Mice were anesthetized prior to vaccination by intraperitoneal (i.p.) injection of 200 μl of ketamine (77 mg/kg of body weight) and xylazine (7.7 mg/kg). Six-week-old CD-1 female mice were vaccinated intranasally with 40 μl of adjuvant only (mock) or whole-cell vaccine (WCV) at day 0 and day 21.

### Cell depletion procedures.

Anti-mouse CD20 MAb (IgG2a, clone 5D2, 250 μg/mouse/dose; Genentech), CD4 MAb (IgG2b, clone GK1.5, BE0003-1, 300 μg/mouse/dose; Bio X Cell), and CD8 MAb (IgG2b, clone 2.43, BE0061, 300 μg/mouse/dose; Bio X Cell) were used for cell depletion trials. Anti-mouse IgG2a (clone C1.18.4, BE0086, 250 μg/mouse/dose; Bio X Cell) and IgG2b (clone Mpc11, BE0085, 300 μg/mouse/dose; Bio X Cell) were used as isotype controls. Antibodies were injected i.p. 2 days before initial vaccination and at day 19 postvaccination (Fig. 1A). Cell depletions were confirmed using flow cytometry (Fig. 1 and 3).

### Bacterial challenge.

To challenge mice, P. aeruginosa PAO1 was grown as described above. Following exponential growth, bacteria were resuspended in PBS (Thermo Fisher; TMS012A), and 20 μl of the challenge dose was administered intranasally at day 35 (2 × 10^7^ to 8 × 10^7^ CFU/dose) (Fig. 1A). In this model, this dose corresponds approximately to the 50% lethal dose (LD_50_) (data not shown). The endpoint for the infection studies was set at 14 h postchallenge to optimize the collection of samples and biological variables while minimizing animal distress. Mice were anesthetized prior to challenge by i.p. injection of 200 μl of ketamine (Patterson Veterinary; 07-803-6637, 77 mg/kg of body mass) and xylazine (Patterson Veterinary; 07-808-1947, 7.7 mg/kg of body mass). Before euthanasia, the surface body temperature of the mice was measured using a noncontact infrared thermometer with a laser sight (Kent Scientific; CODA-IRT) aimed at the abdomen from a distance of 2 in. Mice were euthanized by i.p. injection of pentobarbital (Patterson Veterinary; 07-805-9296, 390 mg/kg of body weight) in sterile 0.9% (wt/vol) sodium chloride (Baxter; 2F124) at 14 h p.c. Cardiac puncture was performed as a secondary method of euthanasia and for blood collection. The lungs of each mouse were weighed and homogenized using Dounce tissue homogenizers with type A pestles (Corning; 7722-7) in 1 ml of sterile PBS. Nasal lavage samples were collected by flushing the nares of each mouse with 1 ml of sterile PBS. Serial dilutions of nasal lavage samples and lung homogenates were plated on PIA plates. Animal experiments were performed according to the *Guide for the Care and Use of Laboratory Animals* (75). The animal protocols used in this study were approved by the West Virginia University Institutional Animal Care and Use Committee (WVU-ACUC; protocol 1606003173).

### Blood collection and tissue preparation for flow cytometry.

Blood samples were collected from the submandibular vein 1 day prior to vaccination and at days 7, 14, 20, and 30 postvaccination. After euthanasia at day 36 postvaccination, blood was collected via cardiac puncture (Fig. 1A). For flow cytometry analysis, blood collection tubes with dipotassium EDTA (BD; 365974) were used to prevent blood coagulation. Red blood cells from the blood samples were lysed using 5 ml of red blood cell lysis buffer (BD; 555899) for 30 min at room temperature. The cells were centrifuged at 1,000 × *g* for 5 min at room temperature and resuspended in 1 ml of RPMI 1640 medium (Thermo Fisher Scientific; A1049101) with 10% (vol/vol) fetal bovine serum (FBS; Gemini Bio; 100-500) twice. The cells were centrifuged again and resuspended in 150 μl of PBS with 1% (vol/vol) FBS.

Spleens and cervical lymph nodes were collected and homogenized using disposable pestles (USA Scientific; 1405-4390) in 500 μl of Dulbecco’s modified Eagle medium (DMEM; Corning Incorporated; 10-013-CV) with 10% (vol/vol) FBS (Gemini Bio; 100-500). Homogenized samples were centrifuged at 1,000 × *g* for 5 min at room temperature. The cell pellets were resuspended with 1 ml of PBS with 1% (vol/vol) FBS and 5 mM EDTA. The cell suspensions were then strained using a 70-μm-pore nylon mesh (Elko Filtering Co.; 03-70/33) with 4 ml of PBS with 1% (vol/vol) FBS and 5 mM EDTA. The samples were centrifuged at 1,000 × *g* for 5 min at room temperature. Red blood cells were lysed in splenocytes using 1 ml of red blood cell lysis buffer and incubated at 37°C for 2 min. The samples were centrifuged at 1,000 × *g* for 5 min and resuspended in 1 ml of PBS with 1% (vol/vol) FBS and 5 mM EDTA.

Single-cell suspensions from blood, cervical lymph nodes, and spleen were incubated with 5 μg/ml of anti-mouse CD16/CD32 Fc block (BD; clone 2.4G2, 553142) for 15 min at 4°C. Samples were then centrifuged at 1,000 × *g* for 5 min at room temperature and resuspended in PBS. Single-cell suspensions were incubated with specific cell surface markers for 1 h at 4°C in the dark. After staining, blood samples were centrifuged at 1,000 × *g* for 5 min at room temperature and washed with PBS twice. The spleen and cervical lymph node samples were fixed with 0.4% (wt/vol) paraformaldehyde overnight. After fixation, the samples were pelleted and washed twice and resuspended in PBS for flow cytometry analysis.

### Flow cytometry.

Lymphocyte populations within the blood, spleen, and cervical lymph nodes were identified using the following anti-mouse antibodies: 1:500 (vol/vol) phycoerythrin (PE)-CF594-conjugated CD45 (clone 30-F11, 562420; BD), fluorescein isothiocyanate (FITC)-conjugated B220 (clone RA3-6B2, 14-0452-82; eBioscience), BV510-conjugated CD3e (clone 145-2C11, 563024; BD), allophycocyanin (APC)-Cy7-conjugated CD4 (clone GK1.5, 552051; BD), peridinin chlorophyll protein (PerCP)-Cy5.5-conjugated CD8α (clone 53-6.7, 551162; BD), and PE-conjugated CD19 (clone 1D3, 553786; BD). Cells were counted using Sphero AccuCount 5- to 5.9-μm beads (Spherotech; ACBP-50-10). Samples were processed using an LSR Fortessa (BD) and analyzed using FlowJo v10 (FlowJo).

### Serological testing by ELISA.

P. aeruginosa-specific antibodies were quantified using enzyme-linked immunosorbent assay (ELISA). Blood was obtained as described above and collected in capillary blood collection tubes (BD; 355967) that were centrifuged for 2 min at 16,200 × *g*. The serum from each mouse was saved in a microcentrifuge tube and stored at −80°C. The lungs were processed as described above, and 360 μl of homogenized lung tissue from each mouse were centrifuged at 16,200 × *g* for 4 min. The lung supernatants were saved in microcentrifuge tubes and stored at –80°C. High-protein-binding 96-well plates (Thermo Fisher Scientific; 15041) were coated with 50 μl/well of 2 × 10^7^ CFU of P. aeruginosa PAO1 in PBS overnight at 4°C. The plates were washed (3 times) with PBS with 0.05% (vol/vol) Tween 20 (Thermo Fisher Scientific; AAJ20605AP) and blocked using 2% (wt/vol) bovine serum albumin (BSA; Research Products International; A30075-250.0) in PBS overnight at 4°C. The plates were rewashed with PBS with 0.05% (vol/vol) Tween 20 (3 times). The serum and lung supernatant samples were serially diluted in plates 1:50 to 1:25,600 and 1:5 to 1:640 using 2% (wt/vol) BSA in PBS, respectively. The serially diluted plates were incubated overnight at 4°C. The antibody titers were detected using goat anti-mouse IgG (SouthernBiotech; 1030-04), IgG1 (SouthernBiotech; 1071-04), IgG2b (SouthernBiotech; 1091-04), IgG2a (SouthernBiotech; 1081-04), IgG3 (SouthernBiotech; 1100-04), IgM (SouthernBiotech; 1021-04), and IgA (SouthernBiotech; 1040-04) conjugated to alkaline phosphatase. The secondary antibodies were diluted in 2% (wt/vol) BSA in PBS. The sample-treated plates were washed (4 times) and incubated with secondary antibodies for 1 h at 37°C. The plates were washed (5 times) and incubated with *p*-nitrophenyl phosphate (PNPP; Thermo Fisher Scientific; 37620) for 30 min in the dark at room temperature. The plates’ signal (optical density at 405 nm [OD_405_]) was detected using a Synergy HTX multimode reader (BioTek). The antibody titers were defined as the highest dilution that yielded an absorbance value two times above the average of blanks.

### Infection with preopsonized P. aeruginosa.

Pools of sera from mice intranasally vaccinated with WCV or curdlan only (mock) were used for the preopsonization experiment. Pooled sera from each group (150 μl) were heat inactivated at 56°C for 30 min and incubated with 2 × 10^8^ CFU of P. aeruginosa PAO1 in 150 μl of PBS in microcentrifuge tubes by tumbling in an end-over-end rotator at 37°C for 30 min. As a nonopsonized control, 10^8^ CFU of P. aeruginosa in 300 μl of PBS were incubated while tumbling in an end-over-end rotator at 37°C for 30 min. After incubation, 30-μl volumes of 10^7^ CFU of P. aeruginosa PAO1 WCV preopsonized, mock-preopsonized, or nonopsonized control were administered intranasally to 5-week-old CD1 mice. The dose for each group was serially diluted in PBS and plated on PIA plates. At 16 h p.c., mice were euthanized as described above. Bacterial burdens in the lungs and nasal lavage samples were determined by plating serial dilutions of the samples on PIA plates.

### Passive immunization with serum.

For passive immunization experiments; the WCV was prepared with Freund’s adjuvant. Mice were vaccinated and boosted with 1 × 10^8^ to 2 × 10^8^ CFU of heat-killed P. aeruginosa PAO1 and 100 μg of complete Freund’s adjuvant (InvivoGen; vac-cfa-10) (WCV-CFA, prime) or 100 μg of incomplete Freund’s adjuvant (InvivoGen; vac-ifa-10) (WCV-IFA, boost) prepared in PBS. The vaccine was adsorbed to the adjuvant by incubation in microcentrifuge tubes by tumbling in an end-over-end rotator for 30 min at room temperature prior to vaccination. Groups of 6-week-old CD1 mice were vaccinated i.p. (200 μl) with WCV-CFA at day 0. On day 21, mice were boosted i.p. (200 μl) with WCV-IFA. As a control, mice were vaccinated with 200 μl of PBS (naive) at days 0 and 21. At day 35 postvaccination, mice were euthanized as described above. Serum was collected by cardiac puncture from a total of 10 mice, and sera from the mice in each group were pooled. A total of 500 μl of pooled WCV or naive mouse serum was administered to 5-week-old CD1 mice via i.p. injection. One hour after serum administration, blood was drawn from the submandibular vein to determine circulating antibody titer and mice were intranasally challenged with 10^7^ CFU/dose of P. aeruginosa PAO1. At 16 h postinfection, mice were euthanized as described above, and blood was collected by cardiac puncture. Nasal lavage samples and lung homogenates, along with their serial dilutions, were plated on PIA plates to quantify viable bacterial burden.

### Statistical analysis.

Prism version 8 (GraphPad) was used for all statistical analyses performed. Data comparisons of more than two groups were performed using one-way analysis of variance (ANOVA) followed by Tukey’s multiple-comparison test for normally distributed data and Kruskal-Wallis test with Dunn’s multiple-comparison test for nonparametric data. One sample *t* test was used to compare the data to the value of the limit of detection. We performed unpaired two-tailed Student’s *t* test (normally distributed) and Mann-Whitney U test (nonparametric) to compare two groups. When calculating the averages of technical and biological replicates, values below the detection limit were recorded as 0. Correlations between two parameters were calculated using linear regression analysis.
